# Production of tailor-made enzymes to facilitate lipid extraction from the oleaginous yeast *Schwanniomyces occidentalis*

**DOI:** 10.1186/s13568-020-00974-z

**Published:** 2020-02-28

**Authors:** Ruud Heshof, Bram Visscher, Eric van de Zilver, Rick van de Vondervoort, Femke van Keulen, Roy J. B. M. Delahaije, Richèle D. Wind

**Affiliations:** HAN BioCentre, HAN University of Applied Sciences, Laan van Scheut 2, 6525 EM Nijmegen, the Netherlands

**Keywords:** *Trichoderma harzianum*, Lytic enzymes, Microbial oil, Fermentation, Disruption

## Abstract

Due to the depletion of fossil fuel resources and concern about increasing atmospheric CO_2_ levels, the production of microbial oil as source for energy and chemicals is considered as a sustainable alternative. A promising candidate strain for the production of microbial oil is the oleaginous yeast *Schwanniomyces occidentalis* CBS 2864. To compete with fossil resources, cultivation and processing of *S. occidentalis* requires improvement. Currently, different cell wall disruption techniques based on mechanical, chemical, physiological, and biological methods are being investigated using a variety of oil producing yeasts and microalgae. Most of these techniques are not suitable for upscaling because they are technically or energetically unfavorable. Therefore, new techniques have to be developed to overcome this challenge. Here, we demonstrate an effective mild enzymatic approach for cell disruption to facilitate lipid extraction from the oleaginous yeast *S. occidentalis*. Most oil was released by applying 187 mg L^−1^ tailor-made enzymes from *Trichoderma harzianum* CBS 146429 against the yeast cell wall of *S. occidentalis* at pH 5.0 and 40 °C with 4 h of incubation time after applying 1 M NaOH as a pretreatment step.

## Introduction

Due to the expanding biobased economy, the interest in microbial oil is increasing (Athenaki et al. 2018; Sitepu et al. 2014a, b). The possibility of economically feasible bio-oil production is being investigated in algae, yeasts, and filamentous fungi (Remmers et al. 2017; Hao et al. 2016; Sitepu et al. 2014b; Ageitos et al. 2011). However, an economically viable production process will require improvements to oil yield through optimization of cultivation and processing, as well as isolation of multiple co-products in a biorefinery setup, indicating the need for mild downstream processing techniques. Efficient extraction of these oils is one of the bottlenecks in ensuring that the use of microbial oil is economically viable (Dong et al. 2016; Ghasemi Naghdi et al. 2016). Oleaginous microorganisms can accumulate more than 20% (w/w) of their total biomass as lipids and are interesting candidates for industrial upscaling (Papanikolaou and Aggelis 2011). One of these oleaginous yeasts is *Schwanniomyces occidentalis,* which is capable of using a broad spectrum of C5 and C6 sugars for the production of microbial oil, mainly as oleic acid (Lamers et al. 2016). In addition, *S. occidentalis* is able to tolerate high concentrations of lignocellulosic hydrolysate inhibitors making it a suitable candidate for growth on renewable lignocellulosic materials (Sitepu et al. 2014a).

Multiple methods have been developed to disrupt the cell wall of oleaginous yeasts and are mainly categorized as chemical, mechanical, physical, and biological methods. These methods can be used on dry or wet biomass, but wet biomass is preferred since it eliminates the costly drying treatment of biomass (Dong et al. 2016). Currently mechanical high-pressure homogenization protocols are used in industry (Athenaki et al. 2018). However, the use of biological methods to disrupt cell walls is a promising technique due to possible prevention of thermal degradation of lipids (Dong et al. 2016). A possible biological technique is the use of enzymes. Enzyme mixtures used for the degradation of fungal cell walls mainly consist of glucanases, chitinases, *N*-acetyl-β-d-glucosaminidases, and proteases, which are commonly found in mycoparasites such as *Trichoderma* sp. (Fan et al. 2014; Yang et al. 2013; Silva et al. 2004; de las Mercedes Dana et al. 2001; Noronha and Ulhoa 2000). *Trichoderma* is therefore often used as a biological control agent in agriculture and in the preparation of fungal protoplasts (Elad et al. 1982). Mycoparasites are grouped in two categories: biotrophic and necrotrophic mycoparasites (Qualhato et al. 2013; Gruber and Seidl-Seiboth 2012). In biotrophic mycoparasitism, multiple organisms benefit from the nutrients obtained at the expense of a target organism, while in necrotrophic mycoparasitism the organism invades and destroys other cells and feeds on the resulting nutrients (Vos et al. 2015; Atanasova et al. 2013). *Trichoderma* sp. are categorized as necrotrophic mycoparasites (Mukherjee et al. 2012). Transcriptomic analysis for *Trichoderma harzianum* and *Trichoderma atroviride* infecting different fungi have revealed that fungal antagonism is a complex system in which many genes are involved related to mycoparasitism (Steindorff et al. 2014; Druzhinina et al. 2011; Reithner et al. 2011; Seidl et al. 2009). These genes are potentially coding for enzymes that are able to disrupt cell walls in yeast.

Biological methods have proven to be successful in the extraction of microbial oil from the yeast *Rhodosporidium toruloides* (Jin et al. 2012). However, a drawback is a thermal pretreatment step required for the yeast in order to weaken the fungal cell wall. In this study we present a production method for tailor-made enzymes (TMEs) that are capable of degrading the cell wall of the oleaginous yeast *S. occidentalis* after a non-thermal pretreatment step. After a NaOH pretreatment of the *S. occidentalis* cells the TMEs can be directly used from the cultivation to disrupt the cell wall.

## Materials and methods

### Strains and culturing

In this study, the strains *S. occidentalis* CBS 2864 and *T. harzianum* CBS 146429 were used. Precultures of *S. occidentalis* were grown at 30 °C in 100 mL yeast-extract peptone dextrose (YPD) medium (10 g L^−1^ yeast extract (Gistex^®^ LS Powder, DSM, the Netherlands), 20 g L^−1^ peptone (Casein Peptone Plus, Organo Technie, France), 40 g L^−1^ glucose monohydrate) using 500 mL shake flasks. For solid plates, an agar solution of 15 g L^−1^ was added. The carbon and nitrogen source were sterilized (121 °C/20 min) separately to prevent Maillard reactions. A spore solution of 1·10^8^ spores mL^−1^ of *T. harzianum* was added to 100 mL potato dextrose broth (CP74.2, Carlroth, the Netherlands) and grown in a 500 mL baffled Erlenmeyer flask in a shaker (New Brunswick™ Innova^®^ 40, Eppendorf, the Netherlands) for 24 h at 150 rpm and 30 °C.

### Bioreactor cultivation

#### Bioreactor parameters and setup

**Fig. 1:**
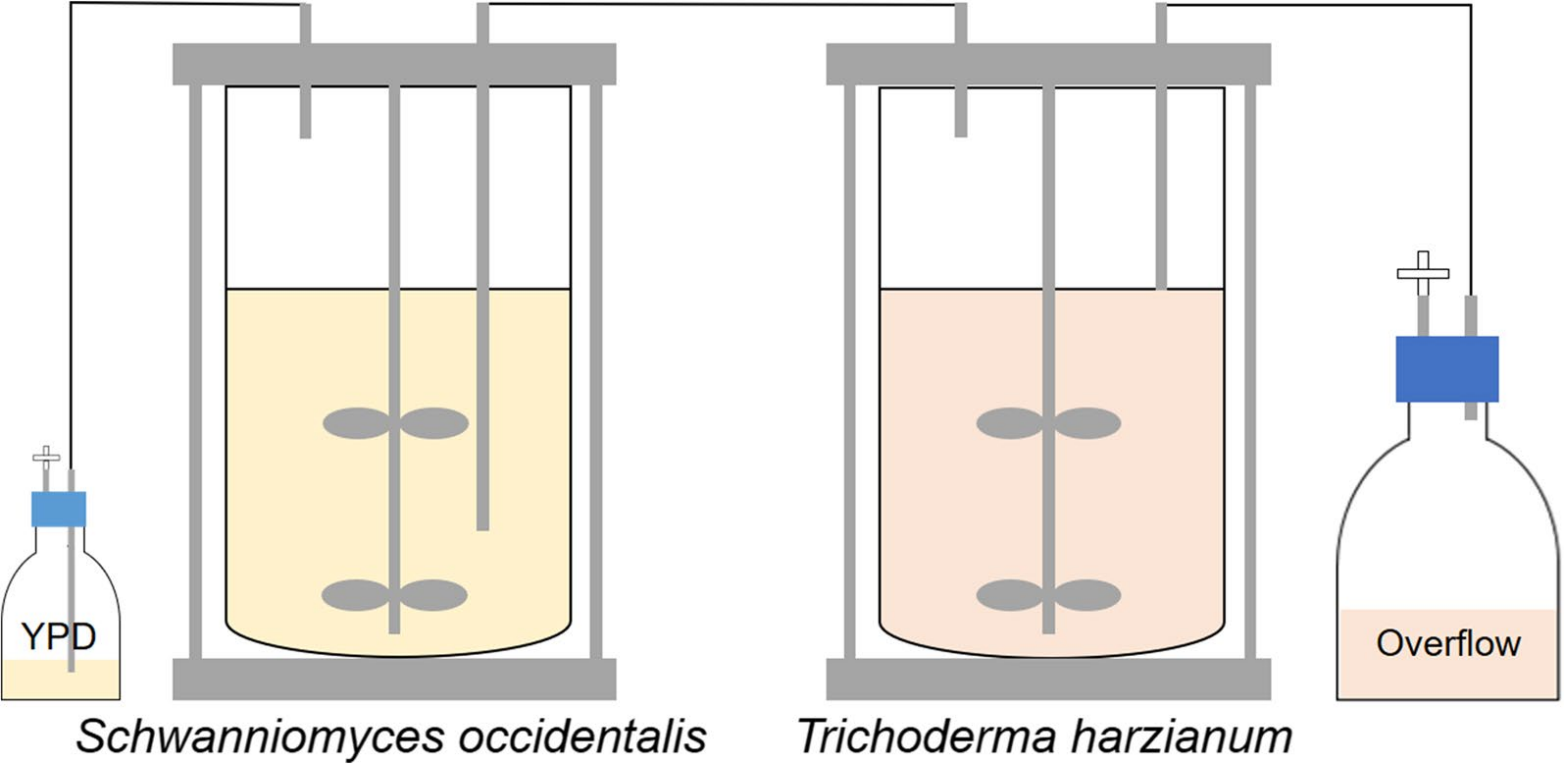
Experimental setup of the double continuous cultivation of the TMEs. Schematic overview of the production of tailor-made enzymes (TMEs) using a double continuous fermentation. In the first bioreactor *Schwanniomyces occidentalis* is continuously grown on YPD medium. In the second bioreactor *Trichoderma harzianum* is fed with living *S. occidentalis*. In this way *T. harzianum* will start producing TMEs against *S. occidentalis*. At the end of the cultivation, the *T. harzianum* broth is harvested and the supernatant separated from the biomass. This supernatant is used as the TME cocktail in further experiments

Precultures of *S. occidentalis* and *T. harzianum* were inoculated in 7 L vessels in a double continuous cultivation setup (Fig. 1). For all the bioreactor experiments BioFlo 115 controllers were used (Eppendorf, the Netherlands). The parameters used for the bioreactor cultivations are shown Table 1. The liquid volume in the *T. harzianum* bioreactor was kept constant at 3.5 L using a Watson-Marlow 120S peristaltic pump (Thermo Fisher Scientific, the Netherlands). The overflow was pumped towards a 4 °C water-cooled bottle to prevent sporulation of the *T. harzianum* biomass. After a cultivation time of 187 h, the cultivation was terminated.

**Table 1:** Parameters used for the bioreactor cultivations of *Schwanniomyces occidentalis* and *Trichoderma harzianum*

Parameter	*S. occidentalis*	*T. harzianum*
Agitation (rpm)	300–1200	300–1200
Dissolved oxygen control (%)	20—agitation cascade controlled	20—agitation cascade controlled
Temperature (°C)	30	30
pH	5.5 ± 0.1	4.9 ± 0.1
Acid	6 M H_3_PO_4_—pump speed 20%	6 M H_3_PO_4_—pump speed 20%
Base	6 M NaOH—pump speed 20%	6 M NaOH—pump speed 20%
Feed	YPD	*S. occidentalis*—pump speed 10%
Gasflow (vvm)	0.5—pressurized air	0.5—pressurized air
Cultivation duration batch phase	18–22 h—until DO and pH started rising	36–40 h—until DO and pH started rising
Cultivation duration fed-batch phase	160–180 h total cultivation time	~ 140–180 h total cultivation time

#### Batch and fed-batch medium

*S. occidentalis* was first grown in a batch phase using the following medium: 20 g L^−1^ glucose monohydrate, 10 g L^−1^ yeast extract (Gistex^®^ LS Powder, DSM, the Netherlands), 20 g L^−1^ peptone (Casein Peptone Plus, Organo Technie, France) and tap water to a total of 5 kg. After the batch phase, *S. occidentalis* was grown continuously using a feed of 2 L YPD medium at a feed rate of 4 mL h^−1^. *T. harzianum* was first grown in a batch phase using medium in which carbon sources, nitrogen sources, and minerals were sterilized (121 °C/20 min) separately to avoid Maillard reactions and precipitation. A carbon source of 12 g L^−1^ glucose monohydrate was prepared in 2 L of demi water. A nitrogen source of 5.6 g L^−1^ yeast extract (Gistex^®^ LS Powder, DSM, the Netherlands), 4 g L^−1^ peptone (Casein Peptone Plus, Organo Technie, France) and 1.2 g L^−1^ urea was prepared in 500 mL of demi water. After sterilization the following stock solutions were added to the bioreactor: 12 mL of 0.2 µm filter-sterilized spore elements (7.20 g L^−1^ MnCl_2_·4 H_2_O, 8.82 g L^−1^ ZnSO_4_·7H_2_O, and 1.25 g L^−1^ CuSO_4_·5H_2_O), 12 mL of a 0.2 µm filter-sterilized 10 g L^−1^ FeCl_3_·6H_2_O solution, 25 mL of autoclaved 3.60 g L^−1^ MgSO_4_·7H_2_O, 25 mL of autoclaved 4.80 g L^−1^ CaCl_2_·2H_2_O, and 250 mL of 1 M K_2_HPO_4_ buffer that was set to pH 5.8 using 1 M of NaH_2_PO_4._ Feeding of *T. harzianum* with *S. occidentalis* was initiated after 19 h at a pump speed of 20 mL h^−1^. After the 187 h of total cultivation time of *T. harzianum,* the biomass was separated from the broth by centrifuging for 30 min at 17,700×*g* at 4 °C. The broth was stored at − 20 °C in aliquots of 500 mL.

#### Sampling

Samples of 5 mL volume from the *T. harzianum* cultivation were taken every 24 h for determination of glucose and protein concentration. To prevent clogging of the sample port by *T. harzianum,* a 0.45 µm filter-membrane (860.300.330, Trace analytics, Germany) surrounded the sample port. The glucose concentration was measured using a d-glucose assay kit (K-GLUC, Megazyme, USA) according to the manufacturer’s instructions. The protein concentration was measured using a Bradford assay with bovine serum albumin as a reference. The protein composition was analyzed by Bolt™ 10% bis–Tris plus SDS-PAGE (NW00100BOX, Thermo Fisher Scientific, the Netherlands).

### Enzymatic activity assay

#### Aliquots of lipid accumulated *S. occidentalis* cells

For activity assays of the TMEs lipid accumulated *S. occidentalis,* cells were grown according to Lamers et al. (2016). The cells were harvested and separated from the supernatant by centrifuging for 5 min at 3894×*g* at 20 °C. The *S. occidentalis* cells were washed with demi water to remove media components and centrifuged for 5 min at 3894×*g* at 20 °C. The *S. occidentalis* pellet was resuspended in demi water until a concentration of 30% (v/v). Aliquots of 3 mL of 30% (v/v) *S. occidentalis* cells containing microbial oil were stored at − 20 °C for further use.

#### *S. occidentalis* pretreatment

A pretreatment step was introduced to weaken the cell wall. Different chemical and physical pretreatment techniques were performed as listed in Table 2. A volume of 3 mL of 30% (v/v) *S. occidentalis* cells containing microbial oil were mixed with 10 mL of the different chemicals. Each mixture was incubated for 24 h at room temperature in a rotary wheel at 20 rpm with a radius of 15 cm. The microwave method of pretreatment was adapted from Jin et al. (2012) and performed with 10 mL of demi water, incubated for 10 min at 700 W. The autoclave pretreatment was an incubation at 121 °C for 20 min. After each pretreatment step the sample was centrifuged for 5 min, 1202×*g* at room temperature. The supernatant was discarded and the pellet was washed with 5 mL of demi water followed by 5 mL of citrate–phosphate buffer (40% of 0.1 M citric acid and 60% of 0.2 M Na_2_PO_4_ to reach a pH of 5.8). The mixture was centrifuged again for 5 min at 1202×*g* at room temperature. The supernatant was discarded and the remaining pellet (~ 1 mL) was diluted with demi water to obtain 30% (v/v) pretreated *S. occidentalis* cells containing microbial oil. Aliquots of 300 μL of pretreated *S. occidentalis* cells containing microbial oil were stored at − 20 °C until required. To further investigate successful pretreatments, a dilution series of 1 M, 0.5 M, and 0.1 M of NaOH was tested.

**Table 2:** Overview of the different pretreatment procedures taken in this study

Sample number	Method/added chemical	Effect
1	1 M NaCl	Osmotic pressure
2	1 M NaOH	Strong base
3	1 M NH_4_OH	Weak base
4	1 M HCl	Strong acid
5	1 M H_3_PO_4_	Weak acid
6	1 M CH_3_COOH	Weak acid
7	Microwave (700 W/10 min)	Heat/radiation
8	1% (v/v) Triton X-100	Detergent
9	1% (v/v) Tween-80	Detergent
10	50 mM DTT	Detergent
11	Autoclaved (121 °C/20 min)	Heat/pressure
12	Demi water + demi water	Negative control
13	Demi water + TMEs	Negative control
14	1 M NaOH + demi water	Negative control
15	1 M HCl + demi water	Negative control
16	Autoclaved (121 °C/20 min) + demi water	Negative control

A volume of 3 mL of 30% (v/v) fat *Schwanniomyces occidentalis* cells was submitted to 10 mL of a chemical listed below. For the microwave, autoclave sterilization, and negative control, 10 mL of demi water was used. Pretreatments were performed for 24 h unless stated otherwise

A 300 µL aliquot of the pretreated *S. occidentalis* cells containing microbial oil were incubated with 50 μL of citrate–phosphate buffer (40% of 0.1 M citric acid and 60% of 0.2 M Na_2_PO_4_ to reach a pH of 5.8) and 900 μL of TMEs to a final protein concentration of 187 mg L^−1^. This mixture was vortexed for 1 min and thereafter incubated at 1000 rpm at 30 °C in a thermomixer (Thermomixer Compact, Eppendorf, the Netherlands) for 4 h and 24 h. The release of oil from *S. occidentalis* was visualized by adding 15 μL Nile red (7726.1, Carl Roth, Germany) solution (1 mg mL^−1^ Nile red dissolved in acetone) to *S. occidentalis* and vortexed for 1 min. Finally, the mixture was centrifuged for 5 min at 2902×*g* at room temperature. Microbial oil released from the cell is then seen as a pink layer on top of the solution.

#### TME activity screening

TME activity parameters were screened using the following factors: temperature (10, 40, and 70 °C), pH (3.0, 5.0, and 7.0), and incubation time (1, 4, and 24 h). The experiment was performed in duplicate resulting in 54 samples. For all samples, 3 mL of 30% (v/v) fat *S. occidentalis* pretreated with 1 M of NaOH was mixed in 3 mL buffer at different pH (3.0, 5.0, 7.0) and 9 mL of TMEs to a final protein concentration of 187 mg L^−1^ of TMEs. The buffers used were 0.1 M glycine–HCl buffer for pH 3.0, 0.1 M citrate buffer for pH 5.0, and 0.1 M phosphate buffer for pH 7.0. To set the pH either 6 M H_3_PO_4_ or 6 M NaOH was used. The tubes were incubated vertically at 250 rpm for 24 h (New Brunswick™ Innova^®^ 40, Eppendorf, the Netherlands). After performing the TME activity assay, the remaining *S. occidentalis* pellet was isolated from the supernatant by centrifugation for 30 min at 3871×*g* at room temperature, and used for oil content analysis using gas chromatography. For the optimization experiments, 0.1 and 0.5 M NaOH as a pretreatment step were used and the experiments were performed as stated above. For the enzyme concentration experiments 1 M NaOH for 24 h was performed as a pretreatment step, and TME concentrations of 187, 93.6, 46.8, 18.7, and 9.36 mg L^−1^ were used for 4 and 24 h of incubation time. Enzyme concentrations were determined via Bradford assay.

### Total lipid analysis of fatty acids by gas chromatography

The remaining oil content in the *S. occidentalis* pellet was measured using gas chromatography by first freeze-drying the cells and methylation of the samples thereafter. As a positive control, a volume of 1 mL 20% boron trifluoride-methanol was added to 30 mg of freeze-dried 30% (v/v) fat *S. occidentalis* cells. A calibration curve was made using pentadecanoic acid (76560-5ML, Sigma Aldrich, the Netherlands). A 100 µL solution of 10 mg g^−1^ heptadecanoic acid (H3500-25G, Sigma Aldrich, the Netherlands) in heptane was added as an internal standard to each sample. A volume of 900 µL heptane was added before the mixture was incubated overnight in a heating block at 70 °C. Thereafter, the samples were cooled to room temperature, 5 mL of demi water was added and centrifuged at 1000×*g* for 10 min at room temperature. The heptane layer was transferred to a fresh vial to which 2 mL of demi water was added, and centrifuged again at 1000×*g* for 10 min at room temperature. A volume of 200 μL of supernatant was added to a gas chromatography (GC) vial. The resulting fatty acid methyl esters (FAMEs) were analyzed by GC in combination with a flame ionization detector. The FAME analysis was performed with a GC-2030 (Shimadzu), which was equipped with a Stabilwax (Restek) column of 30 m × 320 µm × 0.25 µm. The flow of the carrier gas (hydrogen) was set to 2 mL min^−1^ and the injection of 1 µL sample was carried out in split mode (ratio 1:10) at 230 °C. The oven was set to 65 °C for 30 s, then temperature was increased to 220 °C at a rate of 30 °C min^−1^ (held for 60 s) and subsequently increased further to 240 °C at a rate of 10 °C min^−1^ (held for 80 s). The temperature of the detector was set to 240 °C.

### LC–MS/MS analysis

#### Sample preparation

LC–MS/MS analysis was performed for identification of the proteins produced by *T. harzianum*. The proteins were separated by SDS-PAGE and 6 protein bands were excised from the gel for LC–MS/MS analysis. Subsequently, the proteins were reduced by incubating the gel pieces in 10 mM dithiothreitol (DTT) in 25 mM ammonium bicarbonate at 55 °C for 45 min. Then, the proteins were alkylated by incubating the gel pieces in 55 mM iodoacetamide in 25 mM ammonium bicarbonate at room temperature in the dark for 45 min. Finally, the gel pieces were washed with 25 mM ammonium bicarbonate, destained twice with 10% (v/v) acetic acid in 50% (v/v) ethanol at 37 °C for 30 min, and dried in a Savant SpeedVac SPD121P (Thermo Fisher Scientific, the Netherlands) for 10 min.

#### Enzymatic digestion

The gel pieces with the reduced and alkylated proteins were incubated with 0.1 g L^−1^ trypsin in 50 mM ammonium bicarbonate at 37 °C for 24 h. After digestion, the peptides were extracted two times by incubating the gel with 60% (v/v) acetonitrile containing 0.1% (v/v) formic acid at 300 rpm and 37 °C for 1 h. The extracts were combined and stored at − 20 °C until analysis.

#### Analysis

The peptides in the protein hydrolysates were separated using a Dionex Ultimate 3000 LC system (Thermo Fisher Scientific, the Netherlands) equipped with a Zorbax XDB C18 column (4.6 × 50 mm, 1.6 µm particle size; Agilent Technologies, USA). The hydrolysates (10 µL) were injected onto the column thermoregulated at 30 °C. For the separation of the peptides, the following elution profile was used: 0–0.5 min isocratic on 10% B; 0.5–10 min linear gradient from 10 to 90% B; 10–14 min isocratic on 90% B; 14–17 min linear gradient from 90 to 10% B and 17–20 min isocratic on 10% B. Eluent A was milliQ water containing 0.1% (v/v) formic acid and eluent B was 100% MeOH containing 0.1% (v/v) formic acid. The flow rate was 0.3 mL min^−1^. MS data of the hydrolysates were acquired with an online amaZon SL ion-trap MS using HyStar software v3.2 (Bruker Daltonik GmBH, Germany). MS and MS/MS analysis were performed with enhanced resolution as scan mode and ESI in positive mode.

#### Protein identification

Raw MS data were processed with Compass Data Analysis v4.2 (Bruker Daltonik GmBH, Germany) and proteins were identified using BioTools v3.2 (Bruker Daltonik GmBH, Germany) with the Mascot search engine. Peptide sequence matching was first performed against the NCBIprot database without specified taxonomy to check for contamination (e.g. proteins from *S. occidentalis*) and false positives. Peptide sequence matching was then performed against the NCBIprot database with other fungi as taxonomy. Carbamidomethylation of cysteine was set as global modification and missed cleavages by trypsin were excluded. Mass error tolerances for annotation were set to 0.15 Da and 0.5 Da for MS and MS/MS, respectively. Peptides were identified when p < 0.05. The presence of identified proteins was classified as significant with low reliability for proteins originating from *T. harzianum* with ≤ two identified peptides. To analyse the predicted proteins, BLAST and DeepLoc algorithms were used (Altschul et al. 1990; Almagro Armenteros et al. 2017).

## Results

### Bioreactor cultivation

**Fig. 2:**
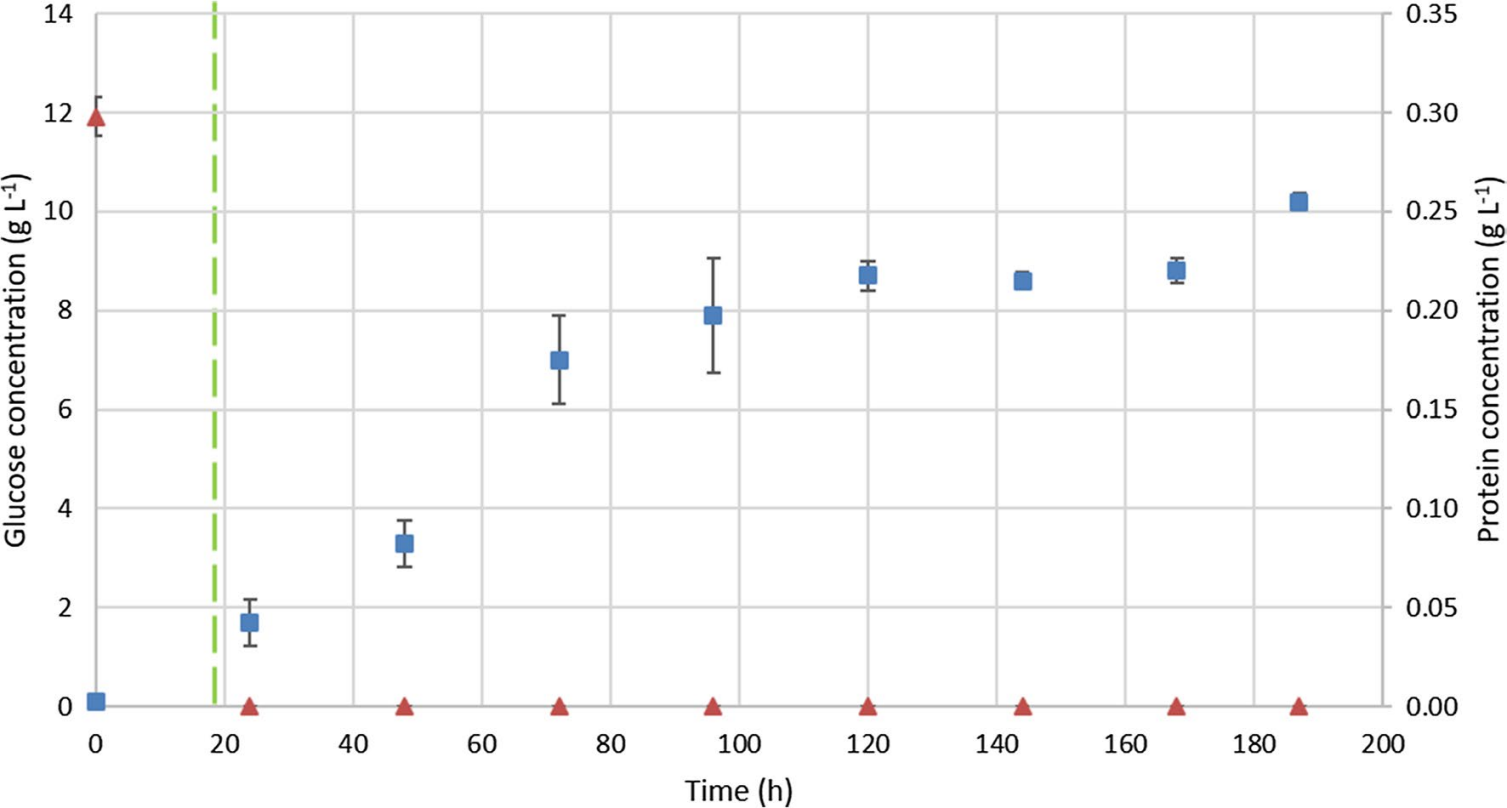
Glucose and protein content analysis of the bioreactor cultivation. Glucose (orange traingle) and protein (blue sqaure) concentration over time during the *Trichoderma harzianum* cultivation. After 19 h the fed-batch phase was initiated using *Schwanniomyces occidentalis* as feed (green dashed line). The glucose concentration remained at 0 g L^−1^ from this time on, while the protein content increased towards 0.26 g L^−1^. After 187 h the cultivation was stopped and the supernatant containing the tailor-made enzymes (TMEs) was harvested

A cultivation procedure using bioreactors was performed to produce tailor-made enzymes (TMEs) by *T. harzianum* fed with wet biomass of non-fat *S. occidentalis* grown on YPD. Every 24 h samples were taken and analyzed for glucose and protein content (Fig. 2). After 19 h the batch phase of *T. harzianum* was completed, since no glucose was present anymore, and the addition of *S. occidentalis* was started by pumping it as a feed into the bioreactor until the cultivation was stopped after 187 h. The glucose concentration remained at 0 g L^−1^ throughout the entire feeding phase with *S. occidentalis*. However, the protein concentration increased during this phase to 0.26 g L^−1^ at the moment of harvesting the supernatant. The resulting broth was stored at − 20 °C until further enzymatic analysis.

### Enzymatic activity assay

**Fig. 3:**
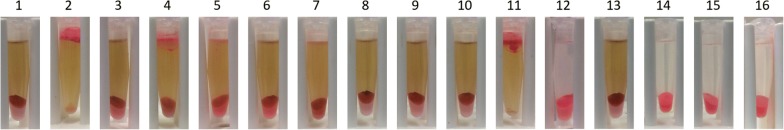
Pretreatment screening analysis. The pretreatment screening analysis exposed the *Schwanniomyces occidentalis* fat cells to chemicals, microwave or autoclaving pretreatment for 24 h prior to addition of the tailor-made enzymes (TMEs). The release of oil was visualized after 24 h of TME incubation time by the addition of Nile red. Sample (1) 1 M NaCl, (2) 1 M NaOH, (3) 1 M NH_4_OH, (4) 1 M HCl, (5) 1 M H_3_PO_4_, (6) 1 M CH_3_COOH, (7) microwave procedure (700 W, 10 min), (8) 1% (v/v) Triton X-100, (9) 1% (v/v) Tween-80, (10) 50 mM DTT, (11) autoclave procedure (121 °C/20 min), (12) control sample with demi water as a pretreatment step and demi water as a lysis procedure, (13) control sample with demi water as a pretreatment step and lytic enzymes as a lysis procedure, (14) control sample with 1 M NaOH as pretreatment step and demi water as a lysis procedure, (15) control sample with HCl as a pretreatment step and demi water as a lysis procedure, (16) control sample by autoclave procedure (121 °C/20 min) and demi water as a lysis procedure

**Fig. 4:**
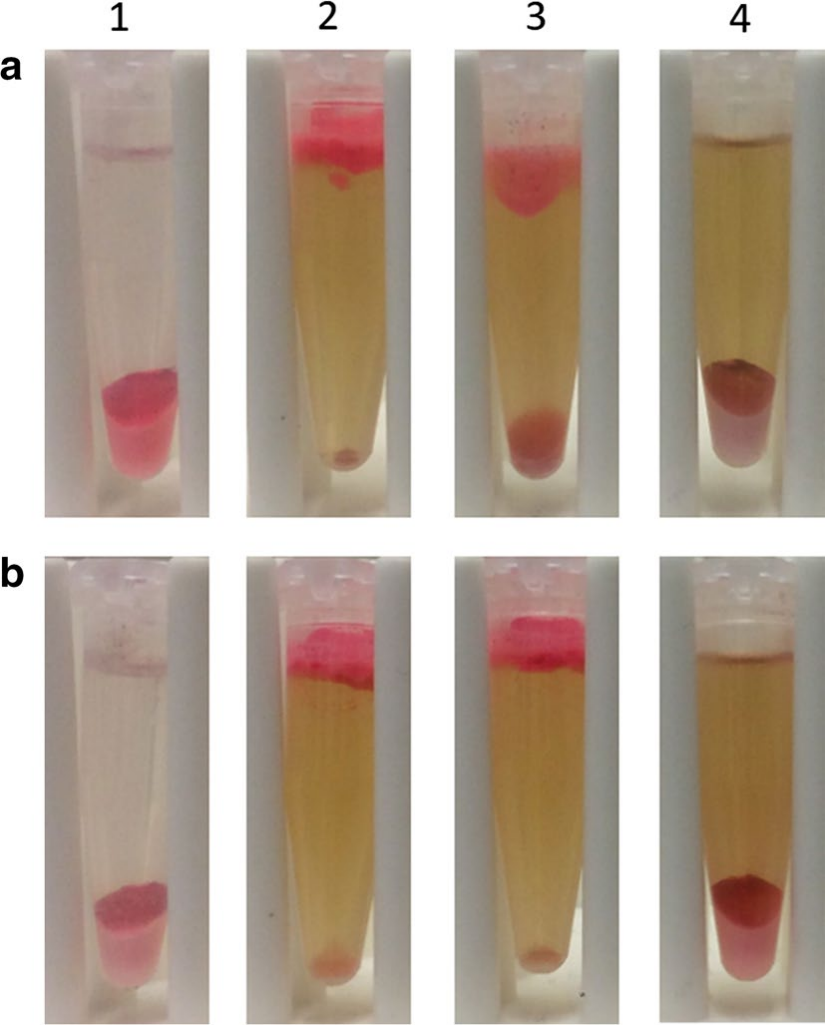
Pretreatment optimization analysis. The pretreatment optimization analysis was achieved by a using different concentrations of NaOH incubated for **a** 4 h with tailor-made enzymes (TMEs) and **b** 24 h with TMEs. Column (1) Control sample incubated with demi water, column (2) 1 M NaOH, column (3) 0.5 M NaOH column (4) 0.1 M NaOH. All assays were performed using a 24 h incubation time of NaOH pretreatment and a final TME concentration of 187 mg L^−1^

**Fig. 5:**
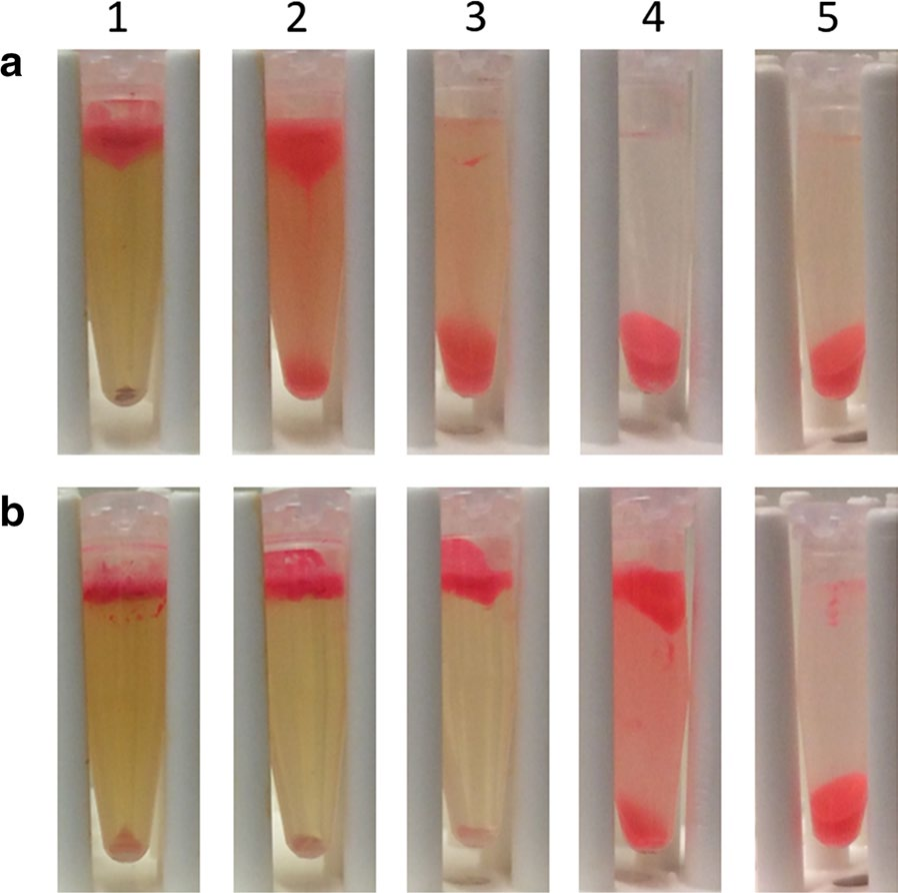
TME concentration analysis. To test the activity of the tailor-made enzymes (TMEs) a dilution was prepared and incubated for **a** 4 h or **b** 24 h. The enzyme concentrations used were column (1) 187 mg L^−1^, column (2) 93.6 mg L^−1^, column (3) 46.8 mg L^−1^, column (4) 18.7 mg L^−1^, and column (5) 9.36 mg L^−1^. The TMEs shown to be active against *Schwanniomyces occidentalis* at a concentration of 93.6 mg L^−1^ with a 4 h incubation time, or at a concentration of 18.7 mg L^−1^ with a 24 h incubation time. However, the best results are obtained when using a TME concentration of 187 mg L^−1^ and 4 h incubation time, or 46.8 mg L^−1^ and 24 h incubation time

After preparation of the TMEs and the lipid accumulated *S. occidentalis* cells, we investigated which pretreatment was most suitable for further experiments. As shown with the Nile red staining, 1 M NaOH was the most promising pretreatment as lipids were released and visible in the upper layer after centrifugation (Fig. 3). One other chemical (1 M HCl) and the autoclavation procedure also reacted positively. However, it was decided to continue with NaOH, since this pretreatment released more oil from the cells compared to pretreatment with HCl (Fig. 3) and is energetically more favorable compared to autoclavation. Next, a concentration series of 1, 0.5, and 0.1 M of NaOH was tested and incubated for 24 h (Fig. 4). Following the incubation, the TMEs were added for 4 h or 24 h. These results showed that the best pretreatments to yield oil were either 1 M of NaOH with a 4 h TME incubation time, or 0.5 M NaOH using 24 h of incubation time with TMEs. It was chosen to continue with the 1 M NaOH pretreatment step because the TME incubation time step is less time consuming. A dilution series of TME concentration showed that the TMEs were active against *S. occidentalis* at a concentration of 93.6 mg L^−1^ at 4 h incubation time, or at a concentration of 18.7 mg L^−1^ at 24 h incubation time (Fig. 5). However, the best results, where there is complete disruption and no oil remains in the pellet, were obtained using a TME concentration of 187 mg L^−1^ and 4 h incubation time, or 46.8 mg L^−1^ and 24 h incubation time.

### Oil content analysis

The TME activity was tested by measuring the oil content in the cell pellets using gas chromatography (Table 3). Unfortunately, released oil on top of the samples could not be measured with reproducible values, since the top layer was fragile and hard to sample in a consistent manner (data not shown). The sample that released the most oil was obtained using the TMEs at pH 5 at 40 °C for 4 h of incubation time. These conditions released all the oil from the cells (0.00 ± 0.00 mg remaining in the pellet). Similarly, using the same conditions but an incubation time of 24 h also released the majority of the oil (0.04 ± 0.05 mg). Positive results were also obtained for pretreatment conditions of pH 3 at 40 °C for 24 h (0.90 ± 0.07 mg), and at pH 5 at 10 °C and 70 °C for 24 h (1.07 ± 0.27 mg and 0.40 ± 0.18 mg, respectively). Based on these results, incubating *S. occidentalis* with TMEs at pH 5 at 40 °C for 4 h is considered the best option due to the mild acidic environment and relatively low temperature and incubation time.

**Table 3:** Total oil content remaining in 30% (v/v) fat *Schwanniomyces occidentalis* pellet after using tailor-made enzymes in a variety of conditions in temperature, pH, and time

Sample	pH	Temperature (°C)	Time (h)	Average oil content in pellet (mg)
1	3.0	10	1	67.59 ± 4.02
2	3.0	10	4	67.44 ± 2.11
3	3.0	10	24	19.69 ± 3.26
4	3.0	40	1	64.61 ± 2.33
5	3.0	40	4	65.82 ± 1.66
6	3.0	40	24	0.90 ± 0.07
7	3.0	70	1	58.22 ± 2.46
8	3.0	70	4	62.12 ± 1.97
9	3.0	70	24	63.27 ± 0.46
10	5.0	10	1	69.58 ± 0.67
11	5.0	10	4	71.50 ± 0.42
12	5.0	10	24	1.07 ± 0.27
13	5.0	40	1	55.02 ± 2.04
14	5.0	40	4	0.00 ± 0.00
15	5.0	40	24	0.04 ± 0.05
16	5.0	70	1	58.82 ± 1.13
17	5.0	70	4	56.89 ± 0.52
18	5.0	70	24	0.40 ± 0.18
19	7.0	10	1	67.05 ± 1.70
20	7.0	10	4	66.17 ± 2.34
21	7.0	10	24	70.48 ± 2.81
22	7.0	40	1	66.57 ± 2.32
23	7.0	40	4	70.64 ± 3.00
24	7.0	40	24	66.49 ± 2.50
25	7.0	70	1	68.59 ± 3.47
26	7.0	70	4	74.30 ± 3.77
27	7.0	70	24	67.90 ± 0.79
Controls—demi water, no enzymes				
28	3.0	70	24	74.91 ± 0.15
29	5.0	70	24	75.07 ± 0.24
30	7.0	70	24	61.24 ± 0.05

The oil content is an average of three technical measurement of a biological duplicate experiment, hence an average of six measurements

### LC–MS/MS analysis

**Fig. 6:**
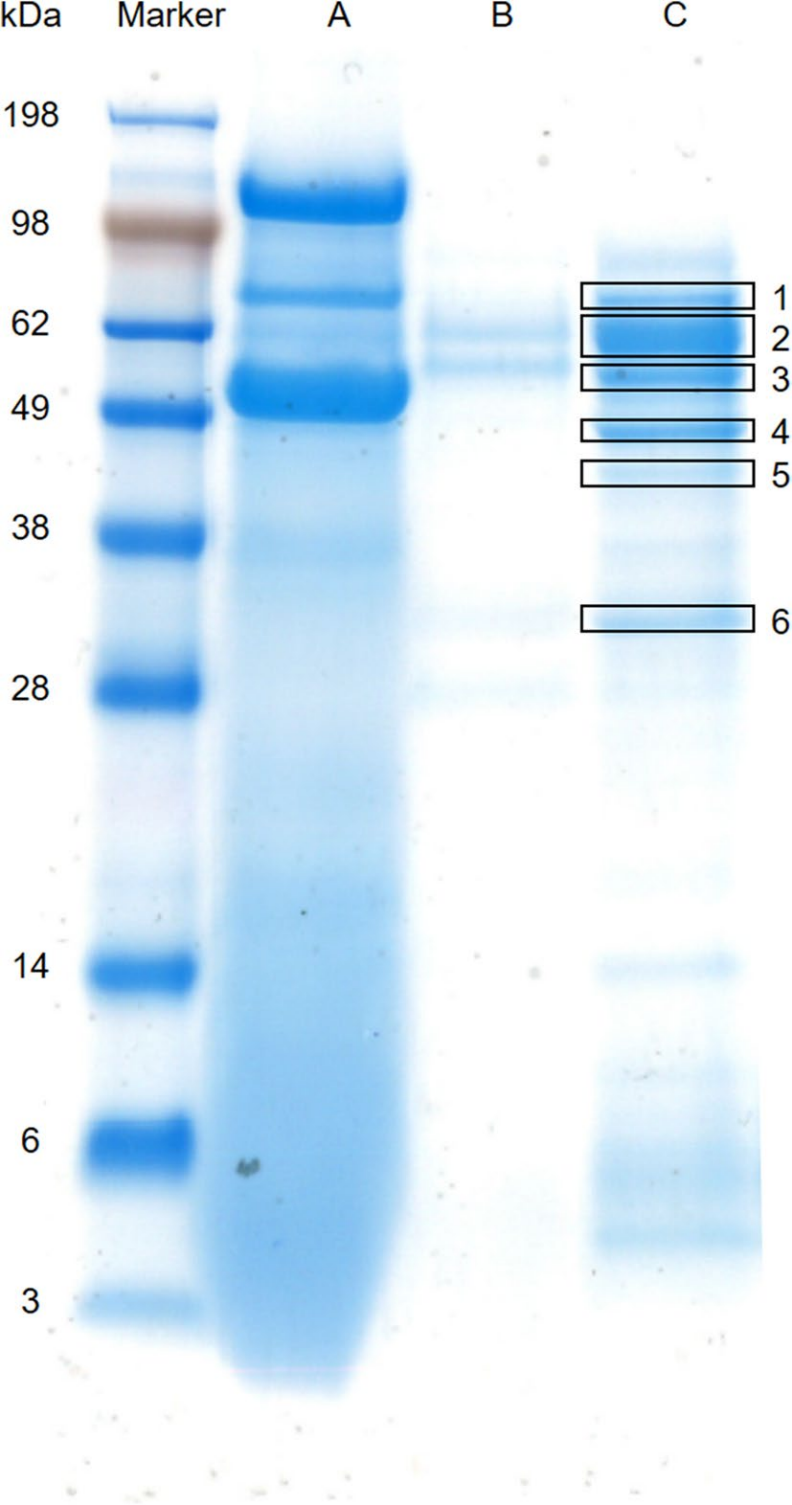
SDS-PAGE analysis of the produced TMEs. SDS-PAGE analysis of the produced tailor-made enzymes (TMEs). Lane Marker) SeeBlue plus 2 pre-stained marker Lane A) YPD medium after *Schwanniomyces occidentalis* growth Lane B) *Trichoderma harzianum* medium before *S. occidentalis* addition Lane C) *T. harzianum* medium at the end of the cultivation. In lane C, the six bands that are boxed were analysed using LC–MS/MS

LC–MS/MS analysis was performed to identify the six main proteins produced by *T. harzianum* (Fig. 6). Two proteins were identified with high reliability; GenBank PKK54770.1 (band 2) and GenBank PKK55157.1 (band 4 and 5), have an amidase- and a sarcosine oxidase-domain, respectively (Table 4). Three other proteins were identified with low reliability (i.e. based on only one peptide hit). These proteins have a serine protease domain (GenBank PKK45954.1; band 1 and 4), a glycoside hydrolase/α-1,2-mannosidase domain (GenBank KKP07817.1; band 3) and a β-1,6-glucanase domain (GenBank ACM42428.1; band 5). All proteins were predicted to be extracellular, with the exception of PKK55157.1, which was predicted to be in the peroxisome. The protein with the lowest molecular weight (± 32 kDa) did not result in a significant hit and remains unidentified.

**Table 4:** Identified proteins and peptides from *Trichoderma harzianum*

Band	GenBank accession code^a^	Protein description	BLAST analysis	DeepLoc prediction	M_w_ based on AA sequence [kDa]	M_w_ based on SDS-PAGE [kDa]	# of peptides^b^	Sequence coverage [%]^b^	Position^b^	Peptide sequence^b^	p^b^
1	*PKK45954.1* *PTB58544.1/XP_02477822.1*	*Hypothetical protein*	*Subtilisin-like serine protease*	*Extracellular*	*± 93*	*± 70*	*1*	*1*	*[668–679]*	*LALQTVTLPAGK*	*6.6·10*^*−4*^
2	PKK54770.1	Hypothetical protein	Amidase	Extracellular	± 61	± 60	4	7	[138–146]	DAGAVLFGK	1.0·10^−3^
	PTB50895.1/XP_024770572.1								[147–15]	AALSEWADMR	9.8·10^−4^
	PNP55812.1								[315–324]	ILTSILTLIK	5.7·10^−4^
	KKO98739.1								[518–527]	YASAIEDLQK	0.025
3	*KKP07817.1* *PKK51292.1* *PNP54665.1* *PTB57154.1/XP_024776831.1*	*Hypothetical protein*	*Glycoside hydrolase family 92 or α-1,2-mannosidase*	*Extracellular*	*± 85*	*± 55*	*1*	*1*	*[247–257]*	*EIPNFNFESVR*	*0.025*
4^#^	PKK55157.1	Hypothetical protein	Sarcosine oxidase	Peroxisome	± 49	± 47	4	15	[31–51]	VVVLEQNNFFNHAGSSNDLAR	4.2·10^−6^
	PTB52097.1/XP_024771774.1								[64–74]	EAMALWDDLER	6.2·10^−3^
									[138–156]	YIGLFAPDNGVINVQLLLR	1.0·10^−5^
									[300–317]	LTSTINPEDIADTQEFIR	0.022
	*PKK45954.1* *PTB58544.1/XP_02477822.1*	*Hypothetical protein*	*Subtilisin-like serine protease*	*Extracellular*	*± 93*	*± 47*	*1*	*1*	*[668–679]*	*LALQTVTLPAGK*	*1.7·10*^*−3*^
5^#^	PKK55157.1	Hypothetical protein	Sarcosine oxidase	Peroxisome	± 49	± 41	3	10	[31–51]	VVVLEQNNFFNHAGSSNDLAR	9.4·10^−3^
	PTB52097.1/XP_024771774.1								[138–156]	YIGLFAPDNGVINVQLLLR	4.8·10^−4^
									[284–291]	IAVDAATR	0.028
	*ACM42428.1*	*β-1,6-glucanase*	*NA*	*Extracellular*	*± 48*	*± 41*	*1*	*2*	*[41–51]*	*FEPALASGITK*	*7.5·10*^*−3*^
	*KKP05604.1*										
	*PKK48835.1/XP_024773174.1*										

These peptides were analyzed via BLAST and DeepLoc prediction to verify whether the protein found via BLAST corresponds to the expected location of the proteins (extracellular). Most proteins were identified to be extracellular, except for sarcosine oxidase (peroxisome)

^#^Number of ^13^C = 1, default: number of ^13^C = 0; *NA* not applicable; Italics indicate protein hits that are significant but with low reliability

^a^Identity between protein hits ≥ 97%

^b^Reported for the first GenBank accession code

## Discussion

The activity of the TMEs show *S. occidentalis* cells can be successfully disrupted and oil can be released (Figs. 4 and 5). The resulting TMEs are a mixture of proteins including proteases, sarcosine oxidases, amidases, mannosidases, and glucanases. It was possible to show that all proteins originate from *T. harzianum*, indicating the absence of contamination from other organisms and degraded proteins from *S. occidentalis* (Fig. 6 and Table 4). Except for β-1,6-glucanase (GenBank ACM42428.1), all identified proteins were predicted based on the sequence data of *T. harzianum* and have been confirmed in this study (Table 4). The hypothetical protein sarcosine oxidase (GenBank PKK55157.1, ± 49 kDa) was identified in band 4 (± 47 kDa) as well as in band 5 (± 41 kDa). Possible reasons may be degradation of the protein due to its extracellular presence instead of the predicted peroxisome, co-diffusion through the gel, or splicing in the corresponding gene (Xie et al. 2015). Chitinase was not detected in the TME mixture. This suggests that either the cell wall of *S. occidentalis* does not contain chitin, or that chitinase is not produced by *T. harzianum* in sufficient quantity to be detected by LC–MS/MS. The latter is more likely as chitin is expected to be present in the cell wall of *S. occidentalis,* based on the robustness of *S. occidentalis* cell wall and the fact that chitin is known to contribute to the strength of cell walls (Arroyo et al. 2016; Lamers et al. 2016). Moreover, chitin is found in many fungal cell walls such as *Candida* sp., and as *Schwanniomyces* sp. are closely related to *Candida* sp. this supports the hypothesis that *S. occidentalis* will have a cell wall comprising chitin (Erwig and Gow 2016; Breuer and Harms 2006). Furthermore, chitinase activity and presence has been identified by previous nLC–MS/MS experiments when grown on chitin-rich medium (Urbina-Salazar et al. 2018). Three different chitinases were identified with a molecular weight of 31, 50, and 82 kDa. It could be that band 6 in Fig. 6 represents a chitinase, since it is within the range of 31 kDa. Unfortunately, the identity of this protein could not be confirmed in this study.

The TME concentration analysis shows the enzyme concentration needed for disruption of *S. occidentalis* cells is dependent upon the duration of TME incubation time; a higher enzyme concentration requires a shorter incubation time (Fig. 5). We expect the enzymes used in this process are the cost-limiting factor and minimizing enzyme concentration will be both economically and practically beneficial for the overall process. Despite these settings, pretreatment with 1 M NaOH is still necessary to disrupt the yeast cells, and is often applied in combination with heat (Safi et al. 2014; Fontaine et al. 2000). It can be concluded that the use of 1 M NaOH as pretreatment in combination with the TMEs makes the heating step redundant (Fig. 4 and Table 3). However, *S. occidentalis* is degraded when used as feed for *T. harzianum* without the use of a NaOH pretreatment (Fig. 6). Consequently, we hypothesize that *T. harzianum* proteins expressed in the cell wall mimic the pretreatment step that is necessary for the extracellular enzymes to function. These proteins could be located in the Papilla-like structures of *T. harzianum*, since many genes coding for both intracellular as well as extracellular proteins are upregulated during mycoparasitism (Atanasova et al. 2013; Druzhinina et al. 2011). It is of interest to identify these proteins and to add them to the TMEs to generate a one-step microbial oil extraction method.

In conclusion, this study demonstrates that microbial oil can be released from *S. occidentalis* using a pretreatment of 1 M of NaOH followed by TME incubation for 4 h at a concentration of 187 mg L^−1^, or using an incubation time of 24 h using 46.8 mg L^−1^ TME concentration. No further treatment needs to be performed with the TMEs themselves and they can be used directly from the cultivation broth. Furthermore, this procedure discards the highly energetically unfavorable pretreatment heating step currently used. These improvements bring microbial oil extraction one step closer towards an economically feasible process contributing to the biobased economy.

## Data Availability

All data is digitally and privately stored via eLABJournal (subscription needed). Enzyme samples for research purposes can be obtained via a material transfer agreement. The strains used in this study are stored at the Westerdijk Fungal Biodiversity Institute (CBS-KNAW).

## Abbreviations

GC: Gas chromatography
DTT: Dithiothreitol
FAME: Fatty acid methyl ester
LC–MS/MS: Liquid chromatography mass spectrometry/mass spectrometry
MS: Mass spectrometry
PDB: Potato dextrose broth
TME: Tailor-made enzyme
YPD: Yeast-extract peptone dextrose
